# Conditioned Medium from Hypoxic Bone Marrow-Derived Mesenchymal Stem Cells Enhances Wound Healing in Mice

**DOI:** 10.1371/journal.pone.0096161

**Published:** 2014-04-29

**Authors:** Lei Chen, Yingbin Xu, Jingling Zhao, Zhaoqiang Zhang, Ronghua Yang, Julin Xie, Xusheng Liu, Shaohai Qi

**Affiliations:** 1 Department of Burns Surgery, First Affiliated Hospital of Sun Yat-Sen University, Guangzhou, Guangdong, P. R. China; 2 Department of Stomatology, The Sixth Affiliated Hospital of Sun Yat-Sen University, Guangzhou, Guangdong, P.R.China; 3 Department of Burns Surgery, The First People's Hospital of Foshan, Foshan, Guangdong, P. R. China; Centro Cardiologico Monzino, Italy

## Abstract

Growing evidence indicates that bone marrow-derived mesenchymal stem cells (BM-MSCs) enhance wound repair via paracrine. Because the extent of environmental oxygenation affects the innate characteristics of BM-MSCs, including their stemness and migration capacity, the current study set out to elucidate and compare the impact of normoxic and hypoxic cell-culture conditions on the expression and secretion of BM-MSC-derived paracrine molecules (e.g., cytokines, growth factors and chemokines) that hypothetically contribute to cutaneous wound healing *in vivo*. Semi-quantitative reverse transcriptase polymerase chain reaction (RT-PCR) and enzyme-linked immunosorbent assay (ELISA) analyses of normoxic and hypoxic BM-MSCs and their conditioned medium fractions showed that the stem cells expressed and secreted significantly higher amounts of basic fibroblast growth factor (bFGF),vascular endothelial growth factor A (VEGF-A) interleukin 6 (IL-6) and interleukin 8 (IL-8) under hypoxic conditions. Moreover, hypoxic BM-MSC-derived conditioned medium (hypoCM) vs. normoxic BM-MSC-derived conditioned medium (norCM) or vehicle control medium significantly enhanced the proliferation of keratinocytes, fibroblasts and endothelial cells, the migration of keratinocytes, fibroblasts, endothelial cells and monocytes, and the formation of tubular structures by endothelial cells cultured on Matrigel matrix. Consistent with these *in vitro* results, skin wound contraction was significantly accelerated in Balb/c nude mice treated with topical hypoCM relative to norCM or the vehicle control. Notably increased *in vivo* cell proliferation, neovascularization as well as recruitment of inflammatory macrophages and evidently decreased collagen I, and collagen III were also found in the hypoCM-treated group. These findings suggest that BM-MSCs promote murine skin wound healing via hypoxia-enhanced paracrine.

## Introduction

The worldwide market for advanced wound care products to promote the repair of hard-to-heal lesions and to reduce the formation of scars consequent to wound healing exceeds $5 billion annually. Thus, wound-healing treatments impose a severe financial burden on the global healthcare system. However, efficacious therapies for the stimulation of recalcitrant wound closure are currently limited [1]–[2].

To this end, abundant experimental evidence has recently demonstrated the therapeutic potential of bone marrow-derived mesenchymal stem cells (BM-MSCs) for the regeneration and repair of damaged tissue in almost all of the major organs of the body, including the heart, brain, lung, liver, kidney, eye and skin [3]. Allogeneic human BM-MSCs are now readily available from commercial stem-cell banks, and autologous BM-MSCs can be harvested from individual patients. Allogeneic and autologous BM-MSCs are both readily expanded *in vitro* without losing their self-renewal capacity. Therefore, BM-MSCs provide an attractive source of cells for tissue repair and regeneration, particularly in cases of tissue damage and loss secondary to an underlying autoimmune disease (e.g., diabetes mellitus type 1 and connective tissue diseases). BM-MSCs also act as seed cells for the production of engineered tissue and as delivery vehicles for gene therapy.

Like other cells utilized for cell-based therapy, BM-MSCs may function through complex interactions with endogenous host cells and tissues via multiple mechanisms of action. The mechanisms by which BM-MSCs ameliorate tissue damage have been subject to debate for years. Currently, two leading theories afford plausible explanations for the therapeutic effects of these cells, as follows: 1) the cell-replacement theory (i.e., BM-MSCs physically and functionally substitute for cells lost to tissue damage) and 2) the paracrine signaling theory (i.e., BM-MSCs activate signal transduction pathways in target cells to encourage tissue regeneration).

The multipotent and self-renewal capacities of BM-MSCs have long been considered advantageous to tissue repair via cell replacement. Further support for the cell-replacement theory is afforded by the observation that BM-MSCs can migrate to sites of injury in response to chemotactic signals *in vivo* [4]. Nonetheless, only a small percentage of grafted BM-MSCs are actually incorporated into, and survive within lesioned tissue [5]–[6], regardless of whether the cells are locally or systemically administered. In some settings, the number of surviving BM-MSCs is, in fact, too low to explain the significant functional improvements observed following cell engraftment. Other studies revealed that transplanted BM-MSCs do not necessarily have to be in close proximity to the damaged tissue to promote wound repair and functional recovery; rather, the BM-MSCs appear to exert their principal therapeutic actions through the secretion of paracrine trophic signals [7].

This study investigated the hypothesis that the proliferation and paracrine function of BM-MSCs are favorably affected by hypoxia so as to enhance their ability to stimulate cutaneous wound healing in mice. This hypothesis was based on the findings that hypoxia-mediated preconditioning of BM-MSCs could reduce hypoxia-induced cell death and increase BM-MSCs proliferation and differentiation [8] *in vitro*. BM-MSCs were cultured in the current report under traditional normoxic (20% atmospheric oxygen (O_2_)) or hypoxic (2% O_2_) conditions. Cell proliferation, as well as the expression of BM-MSC-derived paracrine factors, were then evaluated and compared. Conditioned medium fractions were also collected from normoxic and hypoxic cells, assessed for their content of potentially beneficial cytokines, growth factors and chemokines, and applied to cutaneous wounds in mice. The results of this study now illuminate the considerable influence of oxygenation, and lack thereof, on the biological properties of BM-MSCs, and set forward the intriguing possibility that BM-MSC-derived products might find utility in cell-free therapies to encourage the healing of intractable skin wounds.

## Materials and Methods

### Ethics statement

This study was approved by the Ethics Committee of the First Affiliated Hospital of Sun Yat-sen University (Guangdong, China). The Balb/c nude mice (8–9 weeks old and weighing 20–23 g) employed herein were obtained from the Experimental Animal Center of Sun Yat-sen University. All efforts were made to minimize animal suffering and the number of mice utilized.

### Cell culture

Human BM-MSCs were purchased from ScienCell (Carlsbad, CA, USA). Cells were cultured and expanded as previously described [9].Human CD14+ monocytes were isolated from peripheral blood by using CD14 MicroBeads (Miltenyi Biotec, Auburn, CA, USA) [10]. Murine keratinocytes were isolated from neonatal Balb/c mouse skin via previously established methods [11]. Murine fibroblasts and human umbilical vein endothelial cells (HUVECs) were purchased from the American Type Culture Collection (ATCC, Manassas, VA, USA) and expanded by using a standard cell-culture protocol.

### Induction of hypoxia in BM-MSCs

Hypoxia was induced in BM-MSCs by the use of a sealed chamber that was flushed with a humidified gas mixture composed of 2% O_2_, 5% carbon dioxide (CO_2_) and 93% atmospheric nitrogen (N_2_). The oxygen partial pressure value (pO_2_) in the chamber was controlled and validated with a Compact Oxygen Controller (BioSpherix, Lacona, NY, USA). The hypoxic cell-culture chamber was kept closed throughout the entire experimental procedure.

### DNA assay

A DNA standard was prepared by dissolving salmon testes DNA in TEX (10 mM Tris, 1 mM EDTA, 0.1% Triton X-100), and its concentration confirmed using light at 260 nm from a mQuant UV spectrophotometer (Biotek Instruments, Winooski, VT, USA). Serial dilutions were prepared and used to construct a standard curve for each assay. Cells were washed with PBS twice and then lysed in TEX with 0.1 mg/ml proteinase K (Fisher Scientific, Pittsburgh, PA, USA) at 37°C 1 h. Samples of cell lysate (100 µL) were placed in duplicate or triplicate in to a 96-well plate and 100 µL of Picogreen (Molecular Probes, Eugene, OR, USA) added to each well. The plate was incubated at 37°C for 10 min in the dark and then read on a Packard Fluoro Count fluorescent plate reader (Global Medical Instrumentation, Ramsey, MN, USA).

### BM-MSC proliferation evaluation

BM-MSCs (0.96×10^5^ cells) were seeded into100 mm tissue culture dishes. The cells were incubated in serum-containing complete culture medium or serum-free Minimal Essential Medium (α-MEM) under normoxic (20% O_2_, 5% CO_2_ and 95% air) or hypoxic conditions for 24, 48 and 72 h. The morphology of the BM-MSCs was assessed daily. The cells were detached from the dishes and evaluated at the indicated times by use of DNA assay described above.

### Preparation of conditioned medium fractions derived from normoxic and hypoxic BM-MSCs

BM-MSCs were cultured and expanded under normoxic conditions in serum-containing complete medium. At passage 3–4, the BM-MSCs were seeded into 100 mm tissue culture dishes and allowed to reach ∼80% confluence. The medium was then changed to serum-free α-MEM (5 ml) or other media as indicated, and the cells were cultured under normoxic or hypoxic conditions for another 48 h. Next, conditioned medium fractions were collected from the normoxic and hypoxic BM-MSCs to yield norCM and hypoCM, respectively, centrifuged at 13,000×g at 4°C for 10 min, and stored at −80°C prior to use for enzyme-linked immune sorbent assays (ELISAs), *in vitro* cell proliferation and migration assays, or *in vivo* wound-healing assays. For the *in vivo* wound-healing analysis, aliquots of conditioned media were concentrated by further centrifugation and filtration through a centrifugal filter unit (Millipore, Billerica, MA, USA).

### Keratinocyte, fibroblast and HUVEC proliferation assay

Equal numbers (0.5×10^5^) of keratinocytes and fibroblasts were seeded into 12-well tissue culture plates in vehicle control medium (α-MEM), BM-MSC norCM or BM-MSC hypoCM, HUVECs (0.5×10^5^) were seeded into 12-well tissue culture plates in vehicle control, normoxic or hypoxic BM-MSC-derived conditioned basal endothelial growth medium (EGM-2), and incubated for various periods of time. The media were changed once on day 4 of culture. The cells were detached from the plates and evaluated at the indicated times by use of DNA assay described above.

### Transwell migration assay

A transwell migration assay was performed to assess the chemotactic and migration properties of BM-MSC norCM and hypoCM *in vitro*. Cell suspensions (2×10^5^ CD14+ monocytes, 0.5×10^5^ keratinocytes, 0.5×10^5^HUVECs, or 0.5×10^5^ fibroblasts in 100 µl of medium) were added to the upper chambers of a 24-well transwell plate (pore size for keratinocytes, HUVECs and fibroblasts: 8.0 mm; pore size for CD14+ monocytes: 5.0 mm, Corning Costar, Lowell, MA, USA). Aliquots of vehicle control medium, norCM or hypoCM (600 µl) were then added to the lower chambers. Cells were maintained at 37°C for various periods of time (keratinocytes, 15 h; HUVECs, 8 h; fibroblasts, 24 h; CD14+ monocytes, 2 h). The cells that had not migrated were removed from the upper face of the filters using cotton swabs, cells that migrated from the upper chambers to the lower chambers were then fixed with 4% formaldehyde and stained by 0.1% crystal violet. 3 random ×10 fields were photografted and counted as described previously [12].

### Endothelial cell tube formation assay

Cultured HUVECs (2.5×10^4^ cells per well) were seeded into 24-well plates coated with Matrigel matrix (BD Biosciences, Bedford, MA, USA).Vehicle control, normoxic or hypoxic BM-MSC-derived conditioned EGM-2 (500 µl) was added to each well and incubated with the HUVECs at 37°C in a humidified environment containing 5% CO_2_ for 12 h. Images were captured under a DMI4000B fluorescence microscope (Leica, Solmser Gewerbepark, Germany) at 200× magnification. The number of tubular structures, tubule length, branches and nodes in 4 randomly chosen fields was quantified as previously described with the aid of Olympus Cell R imaging software [13].

### Semi-quantitative reverse transcriptase polymerase chain reaction (RT-PCR)

BM-MSCs (passage 3) were plated at a density of 10,000 cells/cm^2^ in 96-well tissue culture plates and allowed to adhere overnight. After exposure to either hypoxic or normoxic conditions for 48 h, total RNA was prepared from the cells by using TRIzol reagent (Invitrogen, Grand Island, NY, USA) according to the manufacturer's instructions. The SuperScript II First-Strand Synthesis System for RT-PCR (Invitrogen) was used to synthesize cDNA. Relative mRNA expression levels for target genes were determined by using glyceraldehyde 3-phosphate dehydrogenase (GAPDH) as the normalization control. The primers for the target genes are shown in Table 1. Semi-quantitative RT-PCR was performed by using Taq DNA polymerase (New England Biolabs, Ipswich, MA, USA) under the following amplification conditions: denaturation at 94°C for 2 min followed by 30 cycles of 94°C for 30 sec, 56°C for 40 sec, 72°C for 50 sec and, finally, extension at 72°C for 5 min. PCR products were subjected to electrophoresis in 1.5% agarose gels. NIH ImageJ software was used to measure the gray intensity values of the target gene bands, which were normalized to the level of GAPDH gene expression in the same sample.

**Table 1:** Sequence of RT-PCR primers.

Gene	Forward (5′-3′)	Reverse (5′-3′)	Product Size (bp)
GAPDH	GGAGCGAGATCCCTCCAAAAT	GGCTGTTGTCATACTTCTCATGG	197
bFGF	AGTCTTCGCCAGGTCATTGA	CCTGAGTATTCGGCAACAGC	157
IGF	AATTGCCACAAGTCCAGCTG	CAGCCTTGGATGAACGATGG	202
VEGF-A	CTTCTGAGTTGCCCAGGAGA	CTCACACACACACAACCAGG	216
KGF	TTGTGGCAATCAAAGGGGTG	CATTTCCCCTCCGTTGTGTG	170
MCP-1	GCAGCAAGTGTCCCAAAGAA	CTGGGGAAAGCTAGGGGAAA	193
TGF-β1	GTTGAGGAAACAAGCCCAGA	GTTACCCAGGCTGGTCTCAA	199
TGF-β2	CTATCCTGAGCCCGAGGAAG	AGGGTCTGTAGAAAGTGGGC	209
TGF-β3	ACTTGCACCACCTTGGACTTC	GGTCATCACCGTTGGCTCA	114
IL-1β	GGGCCTCAAGGAAAAGAATC	CCCTAGGGATTGAGTCCACA	335
IL-6	GGTACATCCTCGACGGCATCT	GTGCCTCTTTGCTGCTTTCAC	81
IL-8	TTCAGAGACAGCAGAGCACA	AGCACTCCTTGGCAAAACTG	170
IL-10	AAGCCTGACCACGCTTTCTA	GTAGAGACGGGGTTTCACCA	364
TNF-α	AGCCCATGTTGTAGCAAACC	GGAAGACCCCTCCCAGATAG	335
MMP-1	GGTCTCTGAGGGTCAAGCAG	AGTTCATGAGCTGCAACACG	207
Collagen I	TCCAAAGGAGAGAGCGGTAA	GACCAGGGAGACCAAACTCA	693
Collagen III	TTATAA ACCACCCTCTTCCT	TATTATAGCACCATTGAGAC	255

### ELISA analysis

BM-MSC norCM and hypoCM samples were subjected to ELISA analysis for their content of specific cytokines, growth factors and chemokines relevant to wound healing. ELISA kits from R&D Systems (Minneapolis, MN, USA) were used according to the manufacturer's instructions.

### In vivo wound generation and macroscopic examination

Balb/c nude mice were randomly assigned to one of three groups (16 in every group), depending on the type of medium (BM-MSC norCM, hypoCM or vehicle control) applied to the injury site. All mice were anesthetized with inhaled gas anesthesia (O_2_, 2 L/min; isoflurane, 2%) prior to surgery. The dorsal skin was scrubbed with Betadine Veterinary Surgical Scrub and 70% alcohol, and 18 mm round full-thickness excisional wounds were created by using a pair of iris scissors under sterile surgical conditions. The wounds were covered with Tegaderm film (Transparent Film Dressing Frame Style, 3 M Health Care, St. Paul, MN, USA) following topical administration of the medium (100 µl). All animals were housed in individual cages after completion of the surgery. Dressings and medium samples were changed daily for the first 4 days and then removed. Wounds were measured and imaged at 4, 7, 11and 14 days post surgery. Wound contraction was evaluated by gravitational planimetry and expressed as a percentage of the original wound size by using the following formula: (remaining wound size/original wound size) ×100%.

Mice were sacrificed on selected time points via administration of pentobarbital (Euthasol, 250 mg/kg, Virbac Corp., Fort Worth, TX, USA), and the wounds and surrounding tissue were harvested and divided into two parts for immunohistochemical (IHC) or immunofluorescence (IF) analysis, as described below.

### IHC and IF analyses

Skin wound specimens were fixed in 10% formalin at 24°C, followed by dehydration through a graded series of ethanol washes. Dehydrated specimens were embedded in paraffin, cut into 5 µm sections, and mounted onto albumin-coated slides. After dewaxation and rehydration, sections were used for either IHC or IF analysis according to established methods. Briefly, endogenous peroxidase activity was inhibited by immersing the rehydrated sections in 3% hydrogen peroxide (H_2_O_2_) for 10 min. Slides were rinsed with deionized water and placed in an antigen retrieval solution (Target Retrieval Solution, Dako North America Inc., Carpentaria, CA, USA) in a water bath at 98°C to unmask antigens. After a further wash in Tris-buffered saline (TBS) (TBS Automation Washing Buffer, Biocare Medical, Concord, CA, USA), sections were treated with 10% goat serum (Normal Goat Serum, Vector Laboratories, Burlingame, CA, USA) for 1 h to block nonspecific antibody binding. Sections were then incubated overnight at 4°C with the following primary antibodies: rabbit anti-Ki67 monoclonal antibody (1∶400 dilution, Thermo Scientific, Rockford, IL, USA), rabbit anti-CD31 polyclonal antibody (1∶200 dilution, Abcam), rat anti-F4/80 BM8 monoclonal antibody (1∶1500 dilution, eBioscience, Inc., San Diego, CA, USA), mouse anti-collagen Imonoclonal antibody (1∶200 dilution, Abcam) or rabbit anti-Collagen IIIpolyclonal antibody (1∶300 dilution, Abcam). Sections were then washed with TBS and incubated with the appropriate secondary antibody (goat anti-rabbit IgG (1∶200 dilution, Vector Laboratories), goat anti-rabbit IgG H&L (1∶400 dilution, AlexaFluor 568 conjugate, Abcam), or goat anti-mouse IgG (1∶200 dilution, Vector Laboratories)).

For IHC analysis, the reactions were further developed via an avidin-biotin complex reaction with the appropriate reagents (Vector Laboratories). For IF analysis, 6-diamidino-2-phenylindoleindole (DAPI) counter staining was performed, followed by the application of Vectashield Mounting Medium (Vector Laboratories) to the sections to prevent photo bleaching. Labeled cells were visualized via phase-contrast (IHC) or fluorescence (IF) microscopy under a BX51WI microscope (Olympus Korea Co., Ltd.Seoul, Korea). Images of the cells were captured in sections taken through the wound center in order to obtain the maximum wound area for evaluation. The number of positive stained cells and vessels were counted were counted in 4 power fields at 40× magnification as previously described [14]–[16].

### Statistical analysis

All quantitative data are given as the mean ± the standard error of the mean (SEM) for at least three independent experiments. Statistical differences were determined by using Student's t-test or aone-way analysis of variance (ANOVA) followed by Bonferroni's post-hoc test. Differences were considered significant at *p*<0.05.

## Results

### Hypoxia increases the proliferation of BM-MSCs

**Figure 1:**
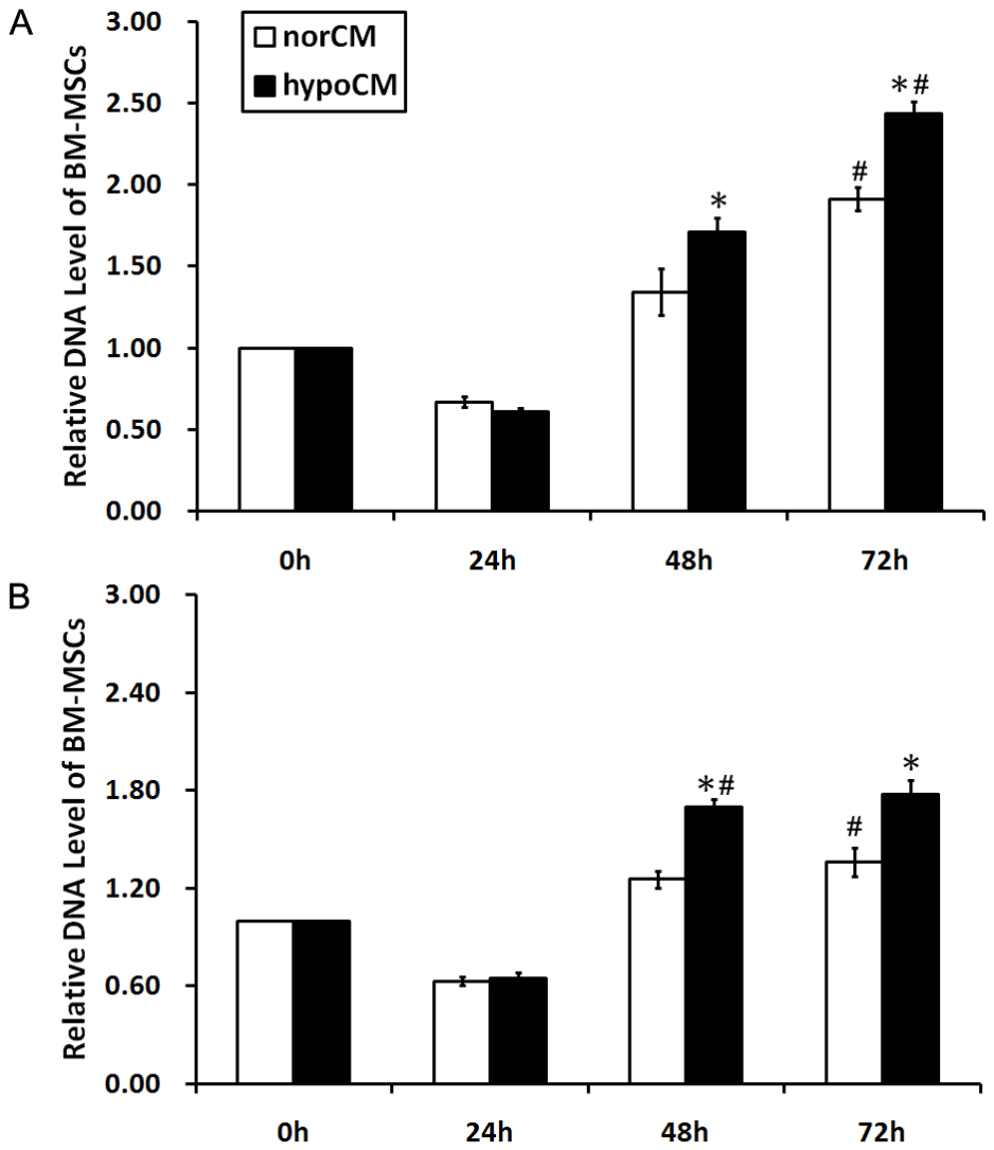
Proliferation of BM-MSCs in serum-containing complete culture medium (A) and serum-free α-MEM (B). Cell proliferation were examined under conditions of hypoxia or normoxia. Data are given as the means± the SEM; *p< 0.05 compared with the norCM group at indicated time points. #*p*< 0.05 compared with indicated earlier time points.

As shown in Figure 1, with or without serum, proliferation of cultured BM-MSCs was significantly increased by hypoxia at 48 h (for BM-MSCs grown in complete culture medium, 1.71±0.09 vs. 1.34±0.15, *p*<0.05; for BM-MSCs grown in serum-free α-MEM, 1.70±0.05 vs. 1.25±0.05, *p*<0.05) and 72 h (for BM-MSCs grown in complete culture medium, 2.43±0.08 vs. 1.91±0.07, *p*<0.05; for BM-MSCs grown in serum-free α-MEM, 1.78±0.08 vs. 1.35±0.09, *p*<0.05).

Proliferation of hypoxic BM-MSCs grown in complete culture medium increased in a time-dependent manner (Fig.1A, *p*<0.05). Similar results were observed when cell proliferation was evaluated in serum-free α-MEM, with the exception of a slight decrease in the proliferative capacity of the 72 h hypoxic incubation group (Fig.1B, 24 h vs. 48 h, 0.64± 0.04 vs. 1.70 ± 0.05, *p*<0.05; 48 h vs. 72 h, 1.70 ± 0.05 vs. 1.78 ± 0.08, *p*<0.05).

### Hypoxia up-regulates the mRNA expression and protein secretion of BM-MSC-derived paracrine factors

**Figure 2:**
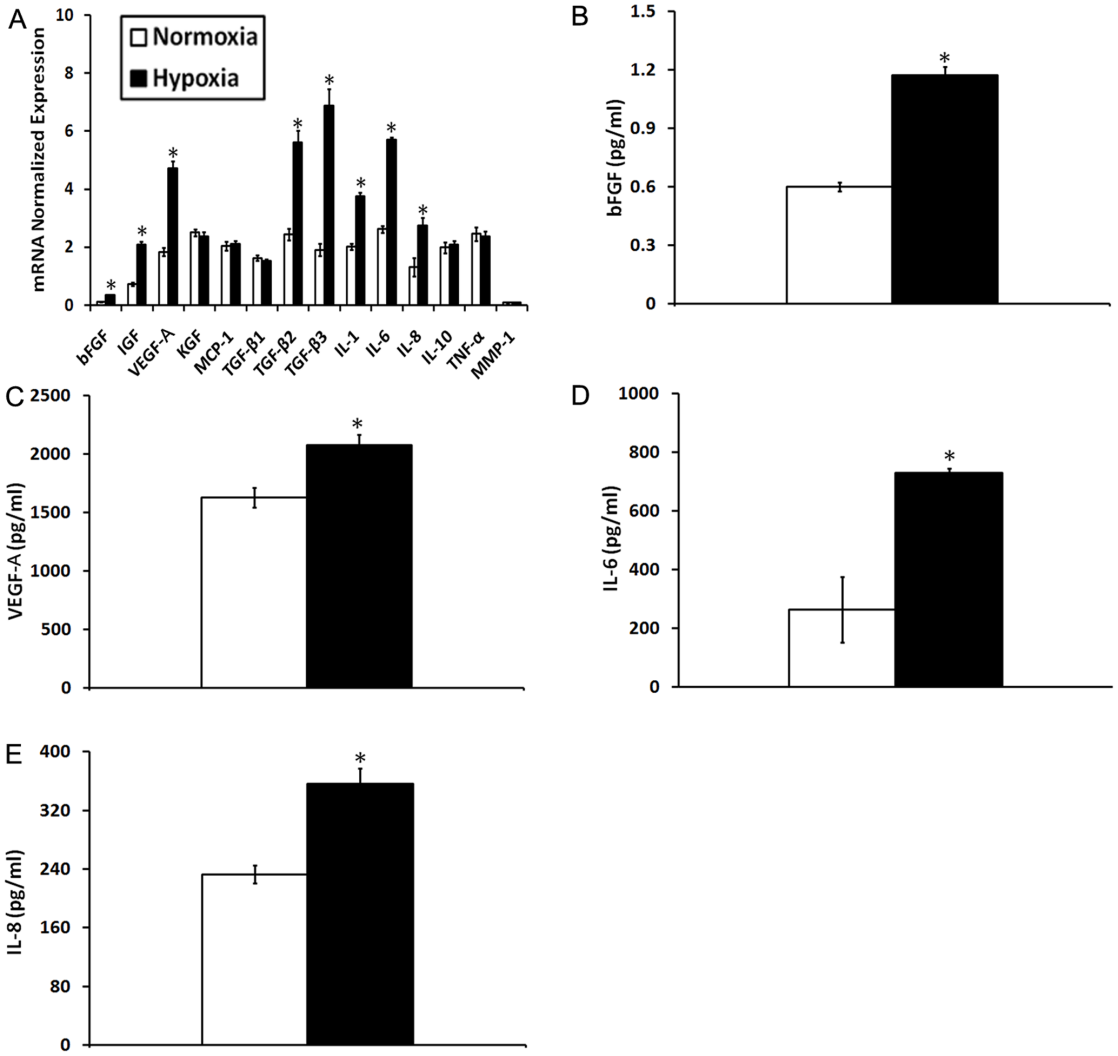
Relative mRNA and secreted protein expression levels of wound-healing-related growth factors, cytokines and chemokines in normoxic and hypoxic BM-MSCs or conditioned medium. (A) RT-PCR assays were performed to measure mRNA levels in BM-MSCs. (B, C, D and E) ELISA assays were performed to measure secreted protein levels in BM-MSC norCM and hypoCM. Data are given as the means± the SEM; *p< 0.05 compared with the expression level of each factor under normoxic culture conditions.

Semi-quantitative RT-PCR analysis was performed to investigate the relative mRNA expression levels of selected growth factors, cytokines and chemokines associated with wound repair in normoxic and hypoxic BM-MSCs. As shown in Figure 2A, hypoxia differentially up-regulated the mRNA expression levels of basic fibroblast growth factor (bFGF, 3.33-fold), insulin-like growth factor 1 (IGF-1, 2.91-fold), vascular endothelial growth factor A (VEGF-A, 2.56-fold), transforming growth factor beta 2 (TGF β-2, 2.30-fold), transforming growth factor beta 3 (TGF β-3, 3.61-fold), interleukin 1 beta (IL-1β, 1.86-fold), interleukin 6 (IL-6, 2.18-fold) and interleukin8 (IL-8, 2.10-fold) (*p*<0.05). On the other hand, similar amounts of keratinocyte growth factor (KGF, 0.95-fold), monocyte chemotactic protein-1 (MCP-1, 1.03-fold), transforming growth factor beta 1 (TGF β-1, 0.93-fold), interleukin 10 (IL-10, 1.05-fold), tumor necrosis factor (TNF-α, 0.97-fold) and matrix metalloproteinases (MMP-1, 1.08-fold) were found in normoxic and hypoxic BM-MSCs (*p*>0.05).

Next, the protein levels of secreted growth factors and cytokines in BM-MSC norCM and hypoCM were further measured and compared by ELISA. In agreement with the semi-quantitative RT-PCR data, bFGF (Fig. 2B, 1.95-fold), VEGF-A (Fig. 2C, 1.27-fold), IL-6 (Fig. 2D, 2.76-fold) and IL-8 (Fig. 2E, 1.53-fold) were all significantly up-regulated in hypoCM vs. norCM (*p*<0.05). However, IGF-1, KGF, MCP-1, TGF β-2, TGF β-3 and IL-1βlevels were similar in the two conditioned medium samples. TGF β-1, IL-10, TNF-α and MMP-1 were even not tested in two conditioned medium samples. The inconsistency between the RT-PCR and ELISA results may be due to relatively low protein expression and/or secretion of these factors under our culture conditions, or the complicated, net collective cell signaling activated by hypoxia.

### Hypoxia enhances mitogenic, chemoattractive and angiogenic paracrine effects of BM-MSCs in vitro

**Figure 3:**
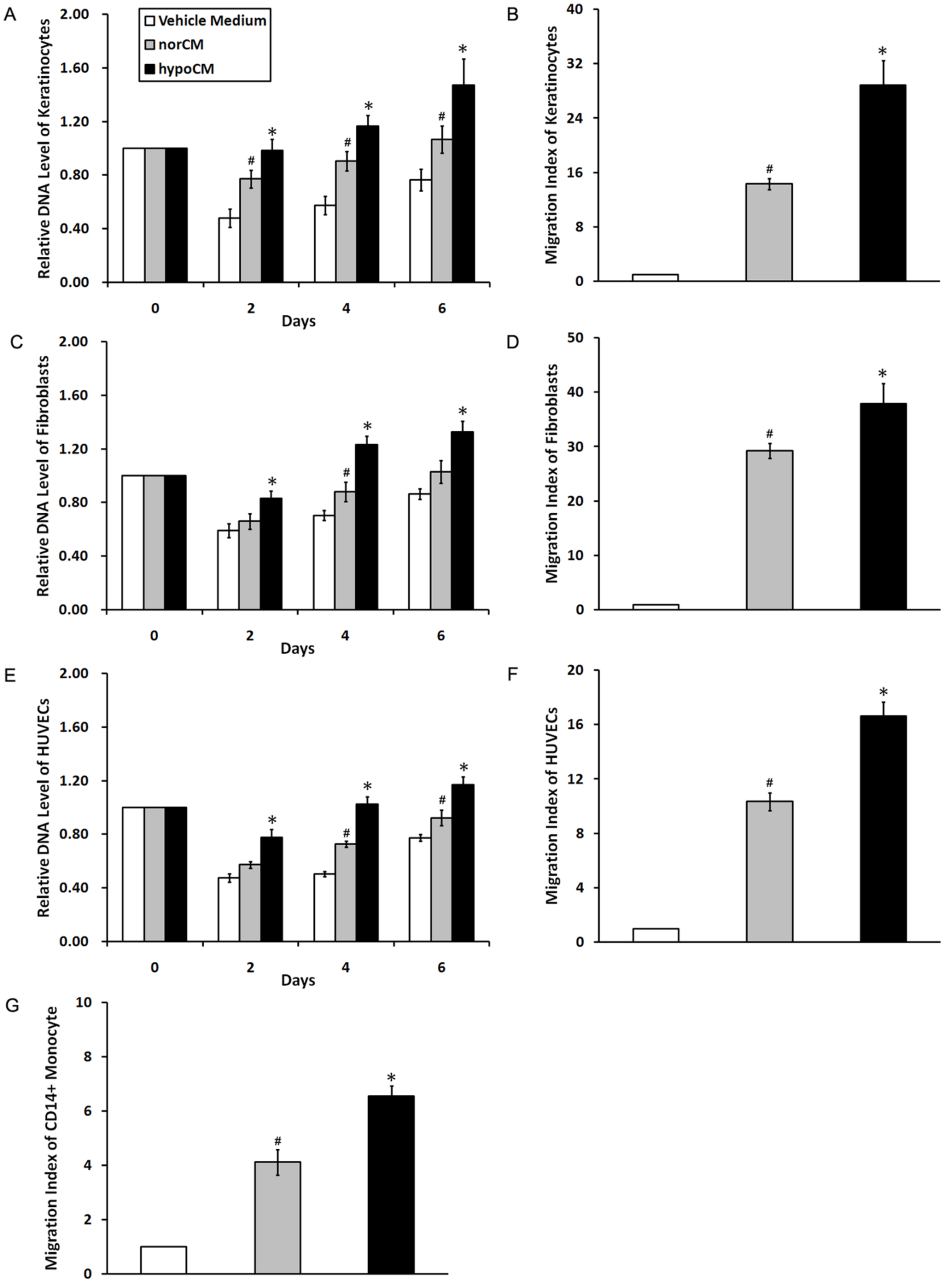
Effects of BM-MSC-derived conditioned medium samples on paracrine cell proliferation and migration. Equal numbers of keratinocytes, fibroblasts and HUVECs were incubated with vehicle control medium, norCM or hypoCM. Cell proliferation was evaluated at indicated time points (A, C and E). Data are given as the means±the SEM; *p< 0.05 compared with the vehicle control or the norCM group. #*p*< 0.05 the vehicle control compared with the norCM group. Equal numbers of keratinocytes, fibroblasts, HUVECs and CD14+ monocytes were added to the upper chambers of 24-well transwell plates, with the indicated medium added to the lower chambers (n = 4 wells per treatment). Cells that migrated to the bottom of the filter were stained and evaluated (B, D, F and G). Data are given as the means± the SEM;*p< 0.05 compared with the vehicle control or the norCM group. #*p*< 0.05 the vehicle control compared with the norCM group.

Cell proliferation, recruitment and angiogenesis are all required for optimal wound repair. Figure 3A, C, E demonstrates that the proliferation of skin cells (i.e., epidermal keratinocytes and dermal fibroblasts) and HUVECs were all substantially increased in the presence of BM-MSC hypoCM relative to norCM or the vehicle control (*p*<0.05). Furthermore, hypoCM exhibited a significantly greater chemoattractive effect than vehicle control medium or norCM on the migration of keratinocytes (Fig 3B, hypoCM vs. vehicle, 28.84-fold; hypoCM vs. norCM, 2.01-fold, *p*<0.05), fibroblasts (Fig 3D, hypoCM vs. vehicle, 37.92-fold; hypoCM vs. norCM, 1.30-fold, *p*<0.05), HUVECs (Fig 3F, hypoCM vs. vehicle, 14.87-fold; hypoCM vs. norCM, 1.44-fold, *p*<0.05) and CD14+ monocytes (Fig 3G, hypoCM vs. vehicle, 6.54-fold; hypoCM vs. norCM, 1.60-fold, *p*<0.05).

**Figure 4:**
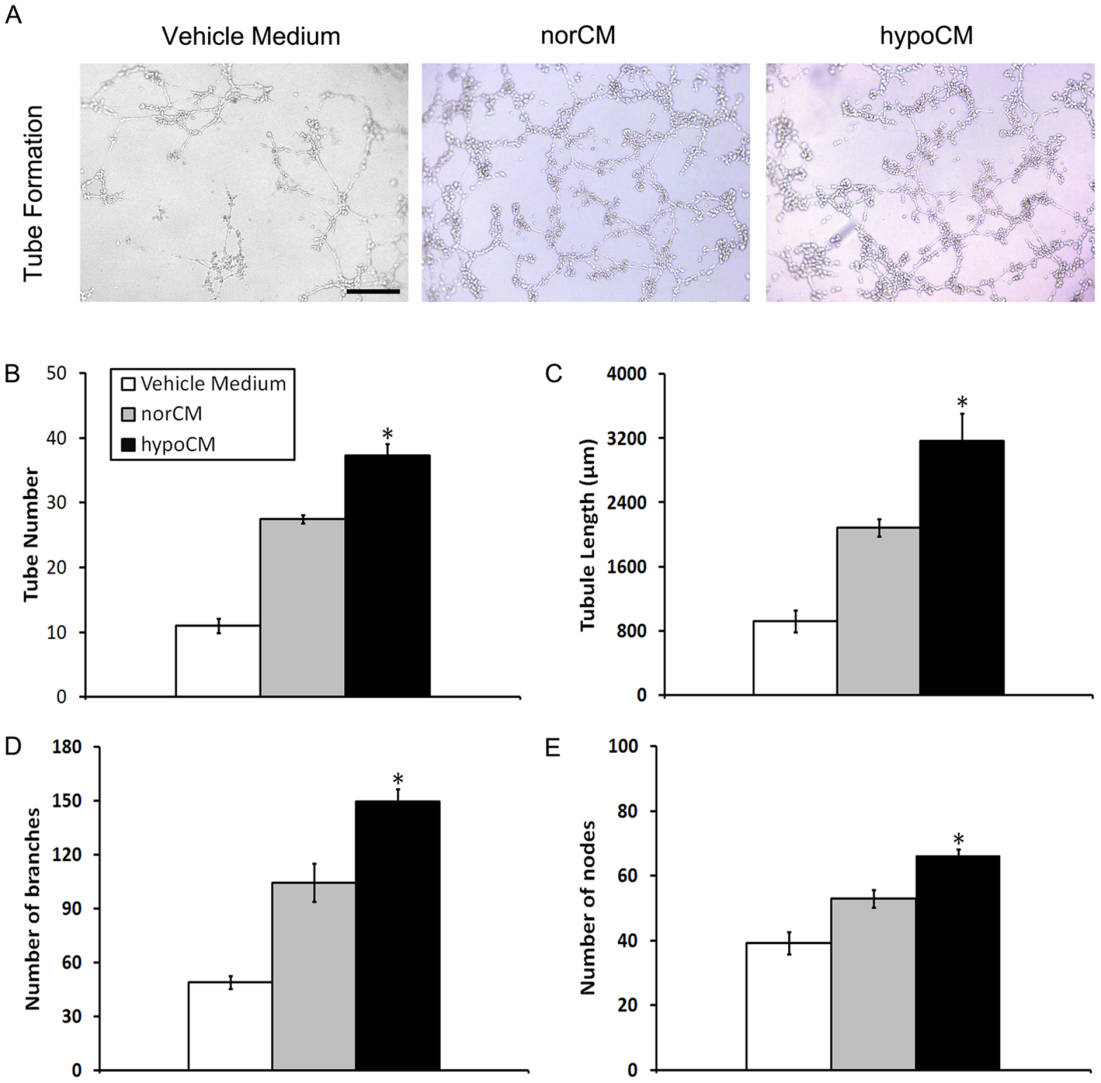
Increased capillary-like tube formation stimulated by BM-MSC hypoCM. (A) HUVECs were seeded onto a Matrigel matrix and incubated with vehicle control medium, hypoCM or norCM for 12 h. (B)Tube formation was quantified. Scale bar, 400 µm for images in (A) (200×). Results are given as the means ± the SEM; *p< 0.05 compared with the vehicle control or the norCM group.

Next, HUVECs were cultured in vehicle control, normoxic or hypoxic BM-MSC-derived conditioned EGM-2 to determine whether hypoxia could augment the contribution of BM-MSCs paracrine to angiogenesis *in vitro*. As a result, BM-MSC hypoCM significantly enhanced HUVEC tube formation ability on a Matrigel matrix compared with the vehicle control or norCM (Figs. 4, *p*<0.05).

### Hypoxia heightens the contribution of BM-MSC-mediated paracrine to cutaneous wound healing in vivo

**Figure 5:**
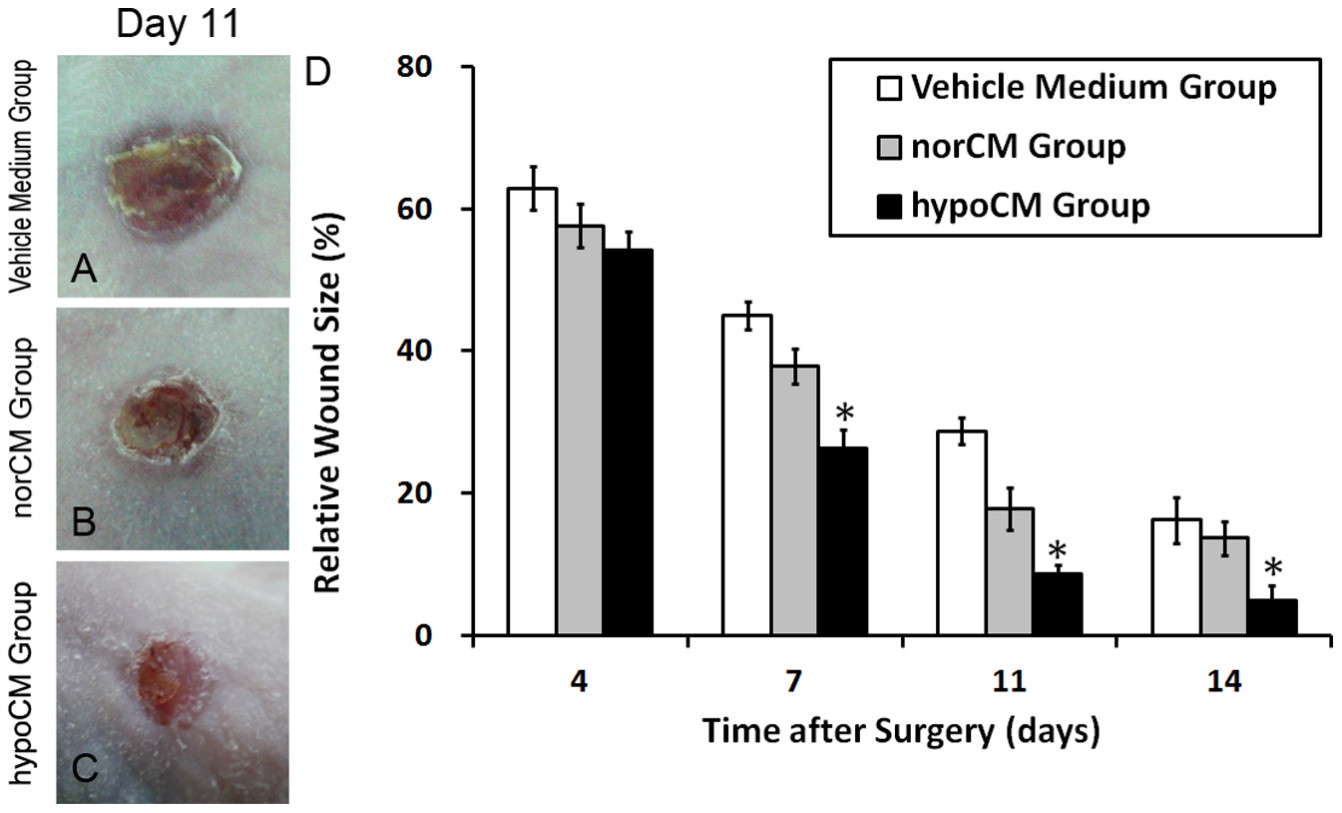
Effects of BM-MSC hypoCM on murine skin wound healing. Representative macroscopic views of cutaneous wounds are shown on day 11 after treatment with vehicle control medium (A), norCM (B) or hypoCM (C). (D) The fraction of the wound area at each indicated time point in comparison to the original wound area was quantified as described in Material and methods, and plotted. Values are givenas the means± the SEM;^*^ *p*<0.01 compared with the vehicle control or the norCM group.

To evaluate the effects of hypoCM on tissue repair *in vivo*, vehicle control medium and concentrated BM-MSC-derived conditioned medium samples (100 µl) were topically applied to Balb/c nude mouse skin wounds. The degree of open, full-thickness excisional wound contraction (indicative of cell migration) was then observed on post-operative days 4, 7, 11and 14. Representative lesions are illustrated on post-operative day 11 (Figs.5A, B, C). BM-MSC hypoCM significantly accelerated wound closure relative to the vehicle control or norCM (*p*<0.05, Fig. 5D), as demonstrated by a significantly smaller wound area in hypoCM-treated mice compared with the vehicle control and norCM groups on post-operative day 7 (26.42±2.48% vs. 45.00±1.97 and 37.92±2.44%, respectively, *p*<0.05). However, the greatest difference in relative wound size between the initial injury and the post-operative, contracted lesion was observed on day 11, when the relative wound size in the vehicle control group was approximately 3.29-fold larger than that in the hypoCM group (28.75±1.83% vs. 8.75±1.25%, *p*<0.05).

**Figure 6:**
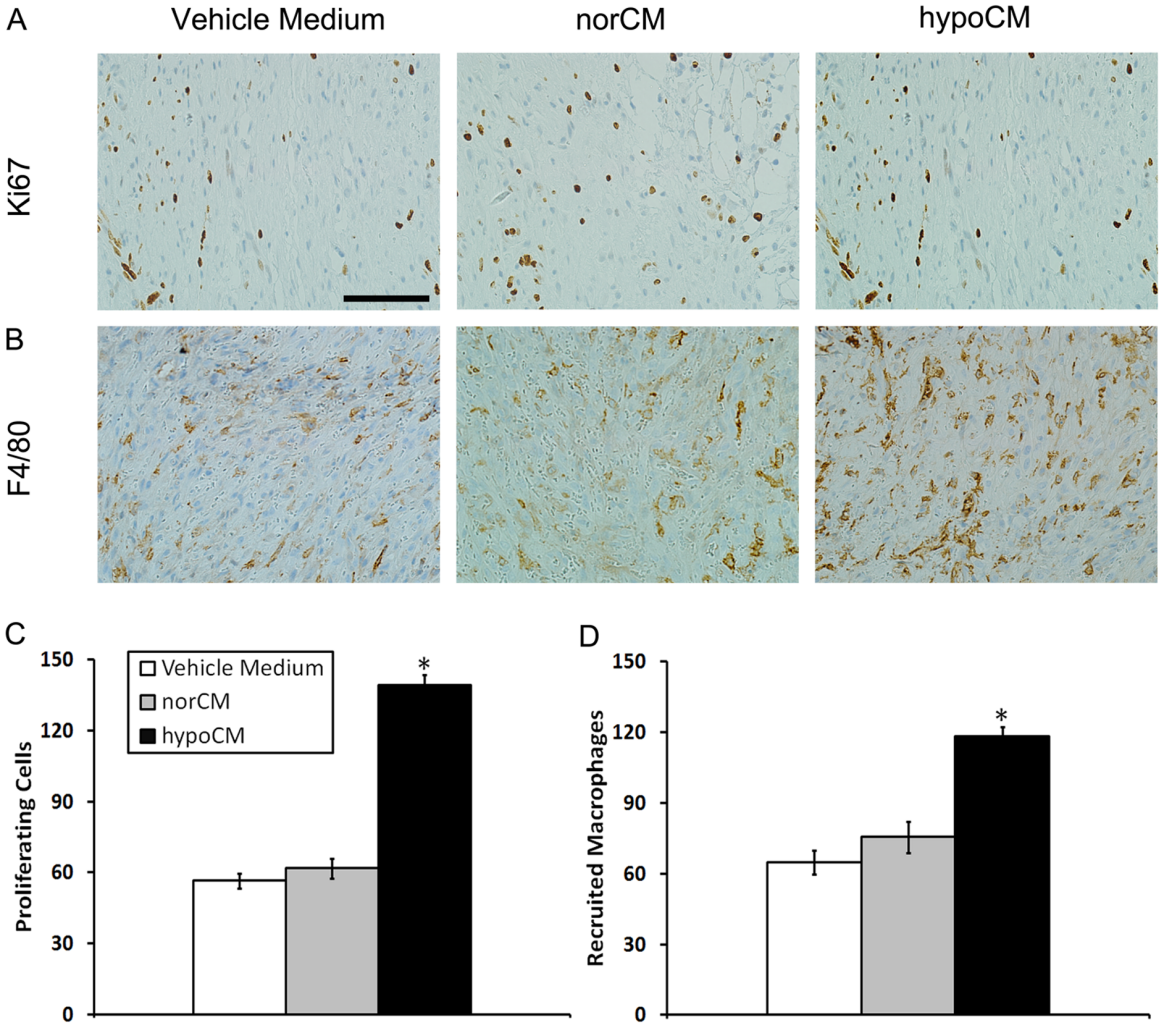
IHC evaluation of wounded mouse skin. Wound sections were evaluated on day 11 by staining with anti-Ki67 and anti-F4/80 antibodies. The numbers of Ki67+ proliferating cells (A, C) and recruited F4/80+ macrophages (B, D) in each of 4 randomly chosen high-power fields in the dermis were counted. Scale bar, 100 µm (400×). Data are expressed as the mean±the SEM;^*^ *p*<0.05 compared with the vehicle control or the norCM group.

The proliferative capacity of the cells within the treated murine skin wounds was next assessed via IHC analysis for Ki67, a nuclear antigen expressed in actively dividing cells (Fig.6A). The number of Ki67+ cells in the dermis of the hypoCM group was significantly increased on day11 compared with the vehicle control and the norCM group (Fig.6C)(139.25 ± 4.31 cells vs. 56.50 ± 3.12 and 61.75 ± 4.13 cells, respectively, *p*<0.05). Taken together, the results of Figures 5, 6A and 6C indicate that hypoxia augments the secretion of pro-migratory and pro-mitotic paracrine factors from BM-MSCs that then target damaged murine skin cells.

Macrophages are generally regarded as beneficial to wound healing, so we examined macrophage recruitment to the treated murine skin lesions by assessing populations of cells positive for F4/80, a marker of mature murine macrophages (Fig.6B). The hypoCM-treated wounds harbored an increased number of F4/80+ cells relative to vehicle control- or norCM-treated wounds (Fig.6D) (118.25 ± 4.03 cells vs. 64.75 ± 5.12 and 75.50 ± 6.70 cells, respectively, *p*<0.05). Because hypoxic and normoxic BM-MSCs express similar levels of MCP-1, this observation suggests that hypoCM contains chemoattractants other than MCP-1, or that hypoCM stimulates host target cells at the injury site to secrete such chemoattractants.

**Figure 7:**
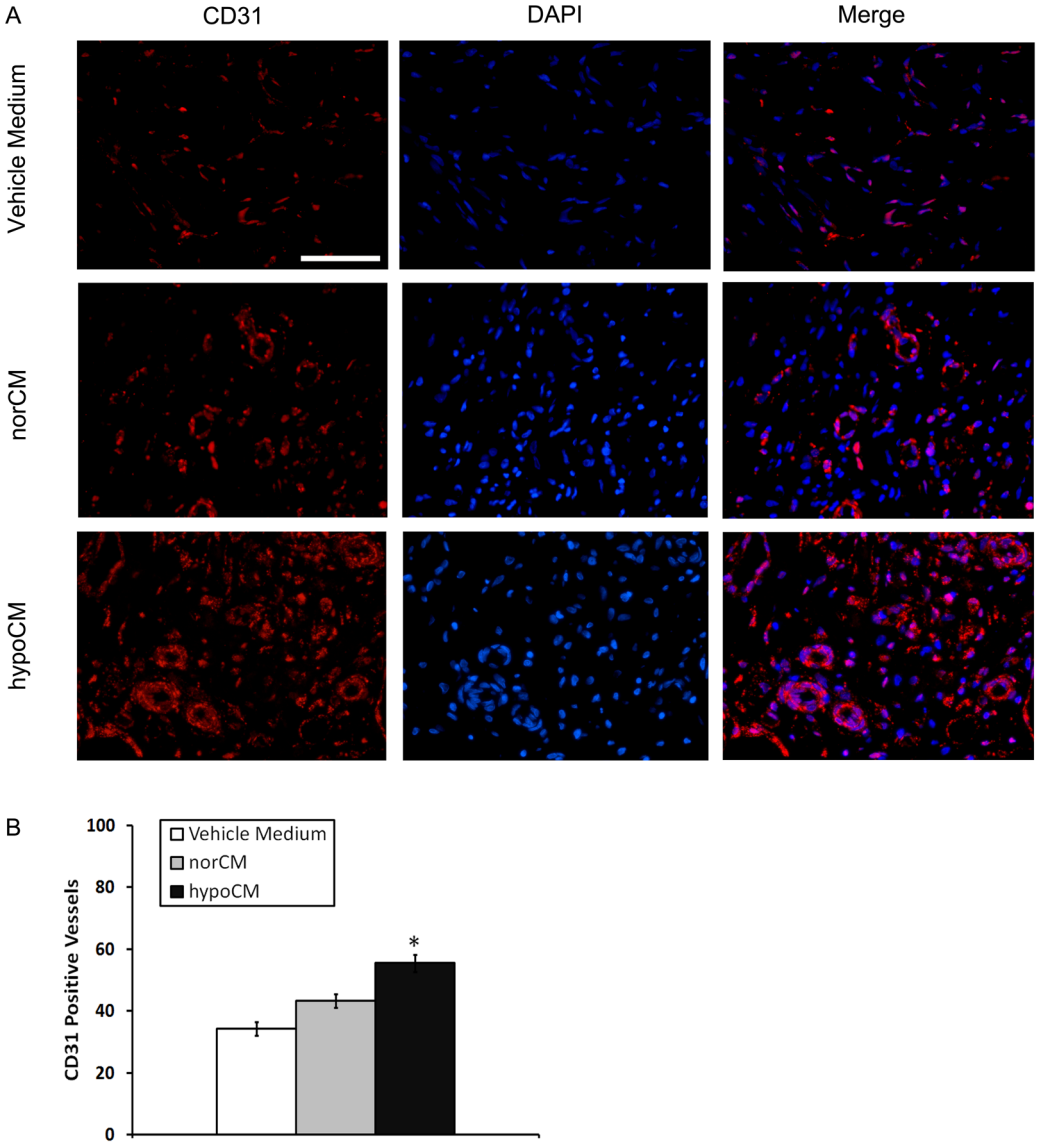
Angiogenesis in the murine skin after wounding. Wound sections were evaluated on day 11 by staining with anti-CD31 antibody. Representative CD31+ vessels are shown (A). The extent of vascularization was determined by assessing the number of CD31+ vessels in each of 4 randomly chosen high-power fields within the injury site (B). Scale bar, 100 µm for images in (A) (400×). Results are given as the means ± the SEM; *p< 0.05 compared with the vehicle control or the norCM group.

As noted above, vascularization of newly formed tissue is an essential component of the wound-healing process. IF staining for the endothelial protein, CD31 (Figs.7A, and B), showed increased blood-vessel formation within hypoCM-treated wounds on day 11 compared with vehicle control- or norCM-treated wounds (55.50 ± 2.78 vessels vs. 34.25 ± 2.25 and 43.25 ± 2.17 vessels, respectively, *p*<0.05). These results are consistent with our *in vitro* data showing that hypoxic BM-MSCs increased their secretion of angiogenic paracrine factors (bFGF, VEGF-A) and enhanced tube formation by HUVECs.

**Figure 8:**
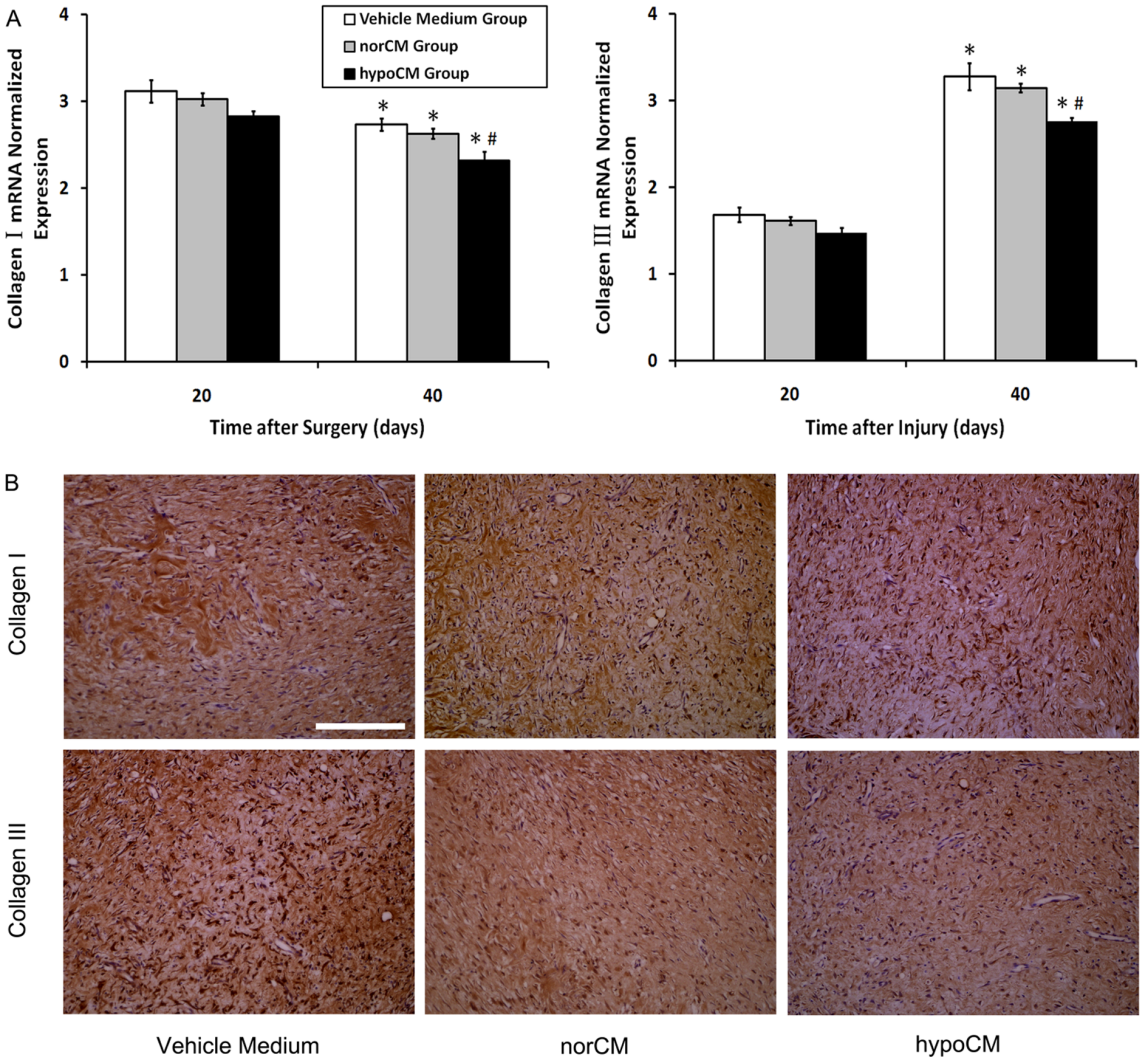
Collagen I and collagen III synthesis analysis. (A). Evaluation of relative collagen I, collagen III mRNA expression. (B) Staining of collagen I and collagen III in scars treated with vehicle control, norCM or BM-MSC hypoCM. Scale bar, 100 µm (400×). Data are expressed as the mean±the SEM; ^*^ *p*<0.05 compared with day 20. #*p*< 0.05 compared with the vehicle control or the norCM group at day 40.

As for the effects of conditioned media on matrix production, we did IHC staining and evaluated the mRNA expression of collagen I and III which constitute the bulk of the scar extracellular cell matrix (ECM). As showed in Figure 8 A, collagen I mRNA expression was reduced overtime (day 20 vs. day 40 for vehicle control group: 1.14-fold, *P*<0.05; for norCM group: 1.15-fold, *P*<0.05; for hypoCM group: 1.22-fold, *P*<0.05) and the decrease of hypoCM group were larger relative to the vehicle control or norCM on day 40 (*P*<0.05). Compared to day 20, collagen III mRNA expression level increased on day 40 (day 40 vs. day 20, for vehicle control group: 1.94-fold, *P*<0.05; for norCM group: 1.95-fold, *P*<0.05; for hypoCM group: 1.87-fold, *P*<0.05) but the increase of hypoCM group were smaller relative to the vehicle control or norCM (*P*<0.05). These results were further confirmed by IHC staining (Figure 8B).

## Discussion

Optimal wound healing requires a well-orchestrated integration of numerous molecular and cellular events that are mediated by growth factors, cytokines and chemokines. Epidermal growth factor (EGF), KGF, IGF-1 and VEGF-A are among the growth factors and cytokines known to enhance normal wound healing [17]-[19]. Consistent with previous reports [20], [21], the current study demonstrated that BM-MSCs expressed and secreted VEGF-A, bFGF and other factors. These growth factors and cytokines function together to modulate the local environment, affecting the proliferation, migration, differentiation and functional recovery of resident cells.

Because of the controversial ability of BM-MSCs to transdifferentiate into cell lineages of all three germ layers, the application of BM-MSC-derived paracrine molecules as a possible therapeutic approach to wound healing is of enormous interest. Such an approach could hypothetically minimize the biological variability of cell-based therapy, overcome questions about cell origin and immunocompatibility, and allow precise dosing with purified paracrine components; thus leading to the development of safe and effective cell-free regenerative strategies with predictable and controllable outcomes. However, traditional *in vitro* culture techniques fail to uphold the stem-cell phenotype of BM-MSCs, and consequently, the achievement of their therapeutic potential has been hindered for years. On the other hand, oxygen tension is now recognized as a crucial component of the stem-cell ‘‘niche”, and the importance of oxygen tension to the maintenance of BM-MSCs proliferative capacity and paracrine mechanisms of reparative action, is currently a matter of lively discussion. Accordingly, the present study focused on the impact of hypoxia on the paracrine functions of BM-MSCs in regard to BM-MSC-stimulated tissue repair.

BM-MSC-mediated paracrine enhances wound healing through three predominant pathways, as evidenced by the demonstrated actions of BM-MSC-derived conditioned medium and secreted molecules: (1) BM-MSC-derived conditioned medium may act as a chemoattractant to recruit specific cell types to the wound, including epidermal keratinocytes, dermal fibroblasts, endothelial cells and macrophages [22], [23]; (2) BM-MSC-derived conditioned medium may regulate cell migration in response to injury [24]; and (3) BM-MSC-derived secreted mitogens may stimulate the proliferation of dermal fibroblasts, keratinocytes and endothelial cells *in vitro* [15], [22], [24], [25,], and possibly *in vivo* as well. Therefore, we turned our attention toward the ability of hypoxia to enhance BM-MSC-elicited paracrine responses in various cell types, both *in vitro* and *in vivo*.

Our current data are consistent with previous reports demonstrating the significantly increased proliferation of BM-MSCs under hypoxic conditions [26]. In addition, semi-quantitative RT-PCR and ELISA data showed that the paracrine function of BM-MSCs (i.e., secretion of selected active factors) toward target skin cells was enhanced by hypoxia, e.g., genes encoding bFGF, IGF-1, VEGF-A, TGF β-2, TGF β-3, IL-1β, IL-6 and IL-8 were all significantly up-regulated in BM-MSCs in response to a low-oxygen environment, and the release of bFGF, VEGF-A, IL-6 and IL-8 was amplified.

Of all the growth factors and cytokines produced by hypoxic BM-MSCs, bFGF and VEGF-A are perhaps the most relevant to wound healing because they can induce angiogenesis, cell migration, proliferation and so on. Our findings regarding bFGF and VEGF-A expression and release are basically consistent with previous investigations [22], [27]. In addition, because their promotive effects on the function of fibroblasts and skin cells migration, IL-6 and IL-8, secreted by BM-MSCs should also involve in the improved wound repair induced by hypoCM. But in the current study, we didn't evaluate if these factors will synergistically effect wound healing. Moreover, pleiotropic cytokine like IL-6 were reported involved in many physiological and pathological conditions. However, it has been proposed as a proinflammatory cytokine involved in a lot of inflammatory diseases [28] or as an anti-inflammatory cytokine involved in the inhibition of T-cell proliferation by MSCs in diseases such as graft-versus-host disease [29]. Considering the results of our *in vitro* and *in vivo* experiments, IL-6 may play a beneficial role in this specific context.

Our recently experiment showed that hypoxia-inducible factor (HIF), a master transcription factor that regulates the expression of hundreds of genes to promote cellular adaptation to hypoxia, pivotally signaled hypoxia-mediated up-regulation of bFGF and VEGF-A secretion in cultured BM-MSCs (data not shown). For future application of CM-BM-MSC or purified factors from conditioned medium in promoting wound healing, pathways involved in enhanced secretion of growth factors and cytokines by hypoxic BM-MSCs still requires more information and further investigation.

We also found that conditioned medium derived from hypoxic BM-MSCs (hypoCM) significantly enhanced the migration and proliferation of keratinocytes and fibroblasts *in vitro*, and the migration of CD14+ monocytes. In contrast, norCM exhibited only modest chemoattractive and mitogenic effects toward these cell types. Also, hypoCM can significantly increase endothelial cells proliferation and migration, promoting early events of angiogenesis. Furthermore, hypoCM significantly accelerated wound closure *in vivo* compared with vehicle control medium and norCM, in addition to angiogenesis, cell proliferation at the injury site, and recruitment of resident macrophages to the wound. It is likely that hypoCM contains elevated levels of HIF-inducible secreted molecules other than bFGF and VEGF-A. These molecules could then act synergistically with bFGF and VEGF-A to enhance the proliferation and recruitment of skin cells after injury, as well as neovascularization.

Type I and III collagens are the central components of ECM products. However, the production of collagen can be a double edged sword: on one hand, it is necessary for wound healing; on the other hand, excess deposition of collagen can result in scarring [30], [31]. Therefore, the appropriate expression of collagen is required for ideal wound healing. Our findings indicate that hypoCM accelerate wound healing by increasing angiogenesis, cell proliferation and recruitment of resident macrophages to the wound, and subsequent regulation of collagen synthesis/degradation as well as alteration of collagen composition at the injury site. This result showed an improved wound repair quality of hypoCM group. But considering the unchanged gene expression lever of MMP-1, and the undifferentiated or low protein expression and/or secretion of TGF-β1-3, this phenomenon may be caused by the alternative and regulating effects of bFGF [32], which in this study been brought up by hypoxia.

In conclusion, the culture of BM-MSC under hypoxic conditions increased their proliferation and paracrine effects to skin cells. These observations indicate that hypoxic BM-MSCs and their secreted products might be employed in regenerative medicine strategies to enhance tissue repair after subcutaneous injury.
