# Correction: Conditioned Medium from Hypoxic Bone Marrow-Derived Mesenchymal Stem Cells Enhances Wound Healing in Mice

**DOI:** 10.1371/journal.pone.0145565

**Published:** 2015-12-18

**Authors:** Lei Chen, Yingbin Xu, Jingling Zhao, Zhaoqiang Zhang, Ronghua Yang, Julin Xie, Xusheng Liu, Shaohai Qi

The authors would like to correct Fig 6, as errors were introduced in the preparation of this figure for publication. In Fig 6A, the hypoCM panel appears as a duplicate of the Vehicle Medium panel. The authors have provided a corrected version of Fig 6 here.

**Fig 6:**
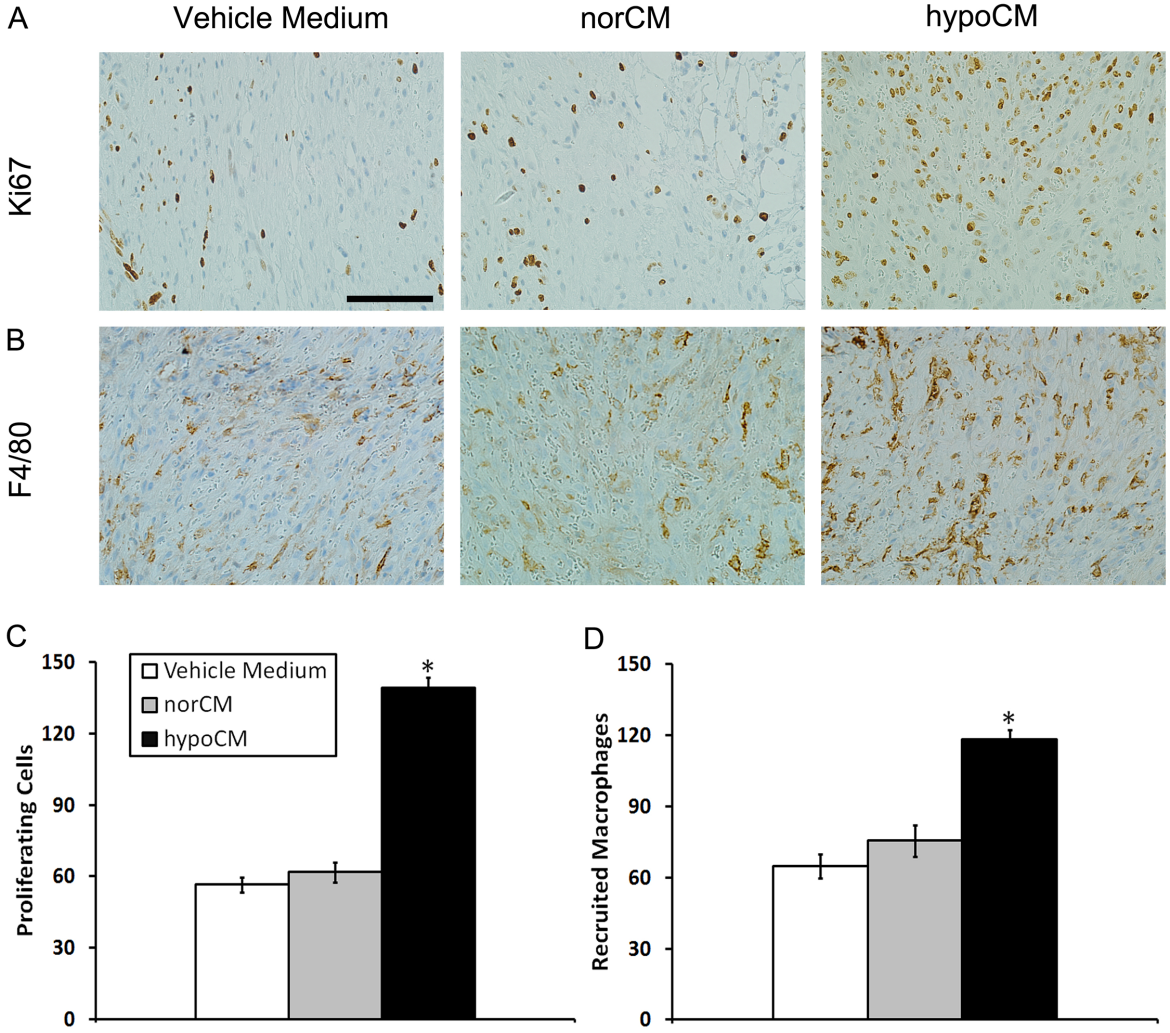
IHC evaluation of wounded mouse skin. Wound sections were evaluated on day 11 by staining with anti-Ki67 and anti-F4/80 antibodies. The numbers of Ki67+ proliferating cells (A, C) and recruited F4/80+ macrophages (B, D) in each of 4 randomly chosen high-power fields in the dermis were counted. Scale bar, 100 μm (400×). Data are expressed as the mean±the SEM;*p<0.05 compared with the vehicle control or the norCM group.

The authors confirm that these changes do not alter their findings. The authors have provided the underlying images as Supporting Information.

## Supporting Information

S1 Fig Ki67 Vehicle Medium. (TIF)

S2 Fig Ki67 norCM. (TIF)

S3 Fig Ki67 hypoCM. (TIF)

S4 Fig F4/80 Vehicle Medium. (TIF)

S5 Fig F4/80 norCM. (TIF)

S6 Fig F4/80 hypoCM. (TIF)
