# Supplementary figures and images for: Correction: Conditioned Medium from Hypoxic Bone Marrow-Derived Mesenchymal Stem Cells Enhances Wound Healing in Mice
Source: PLoS One. 2015 Dec 18;10(12):e0145565. doi: 10.1371/journal.pone.0145565 (PMC4684322; doi:10.1371/journal.pone.0145565)

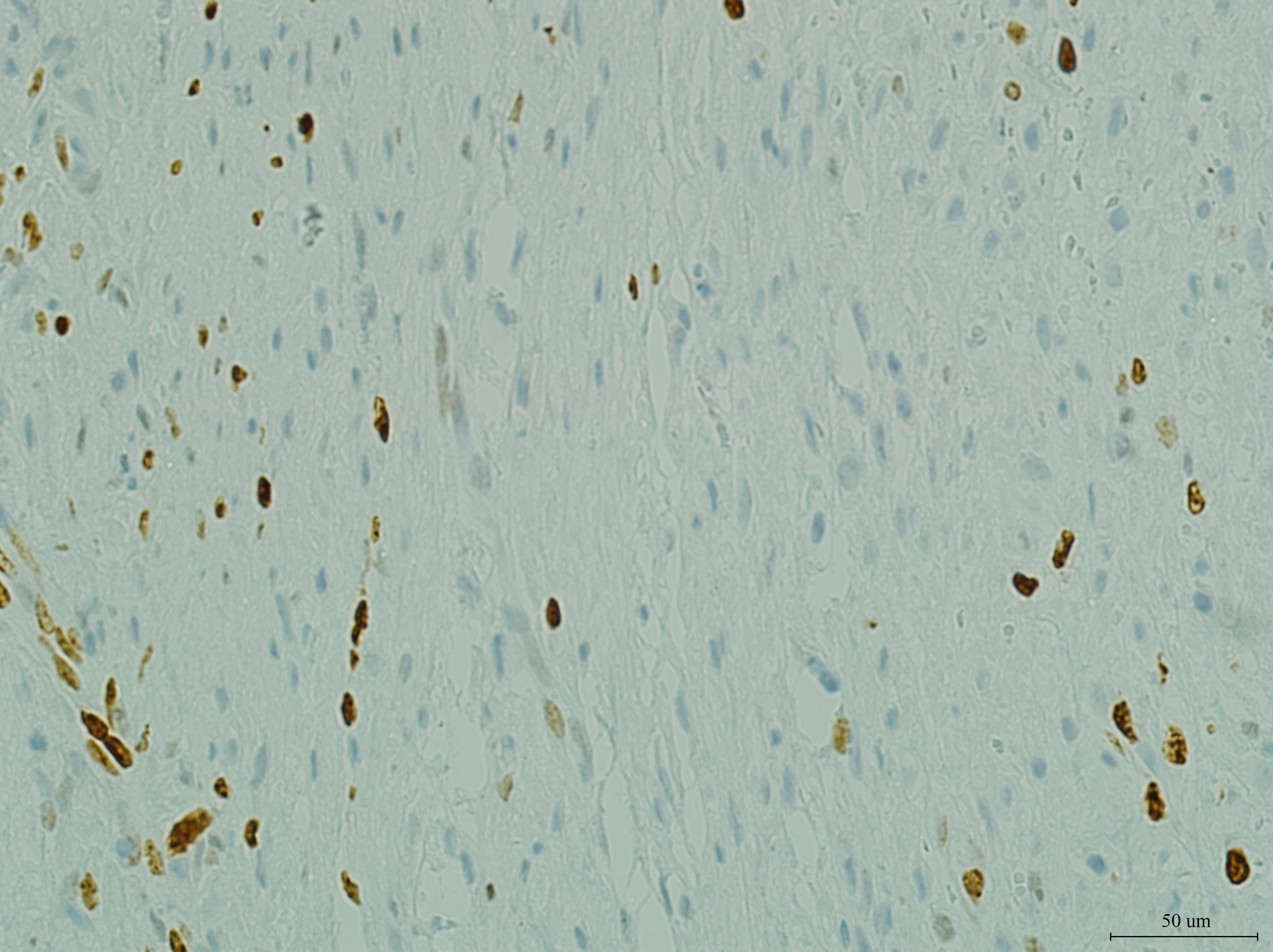

Supplement: S1 Fig — (TIF) [file pone.0145565.s001.tif]

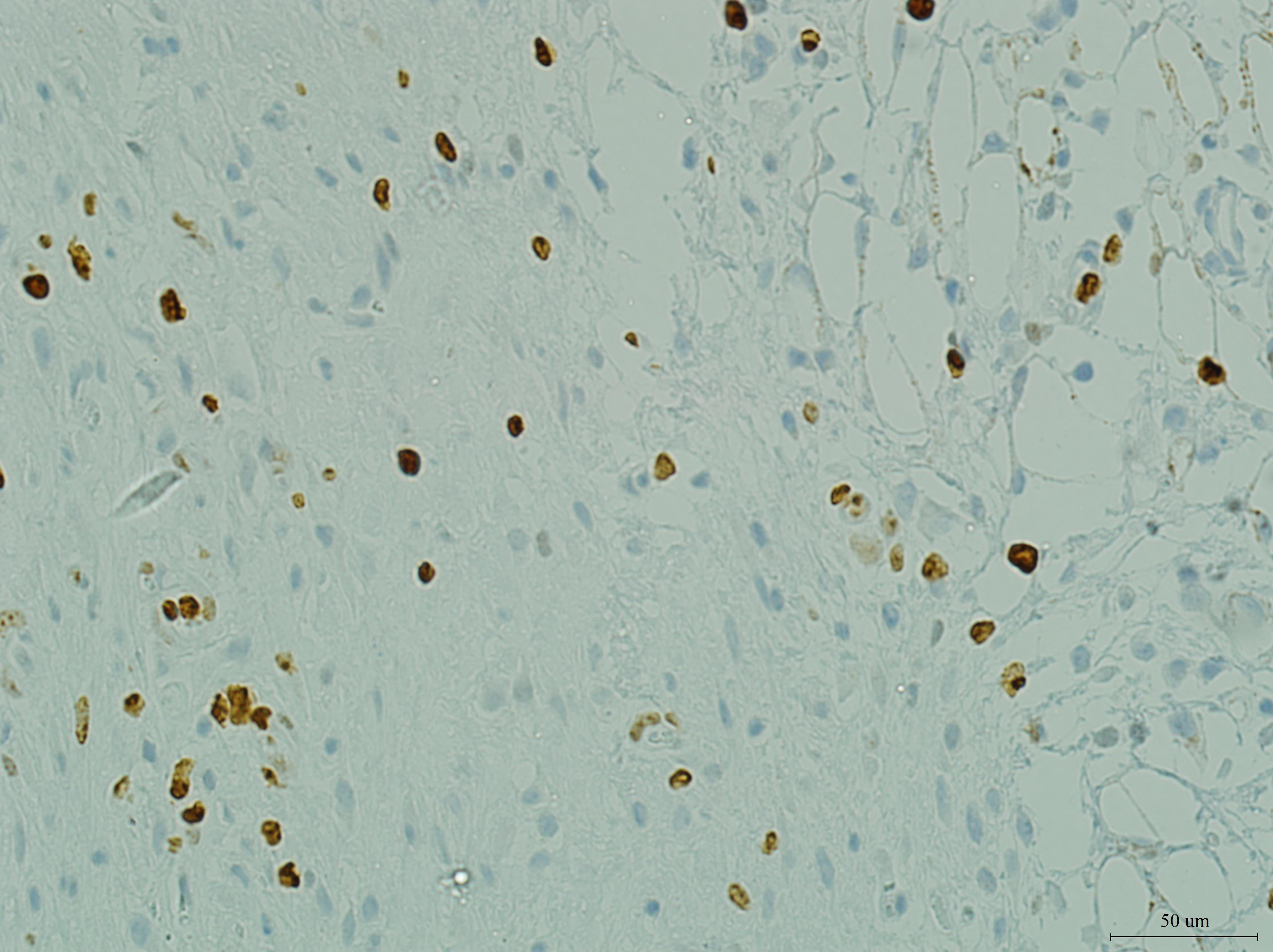

Supplement: S2 Fig — (TIF) [file pone.0145565.s002.tif]

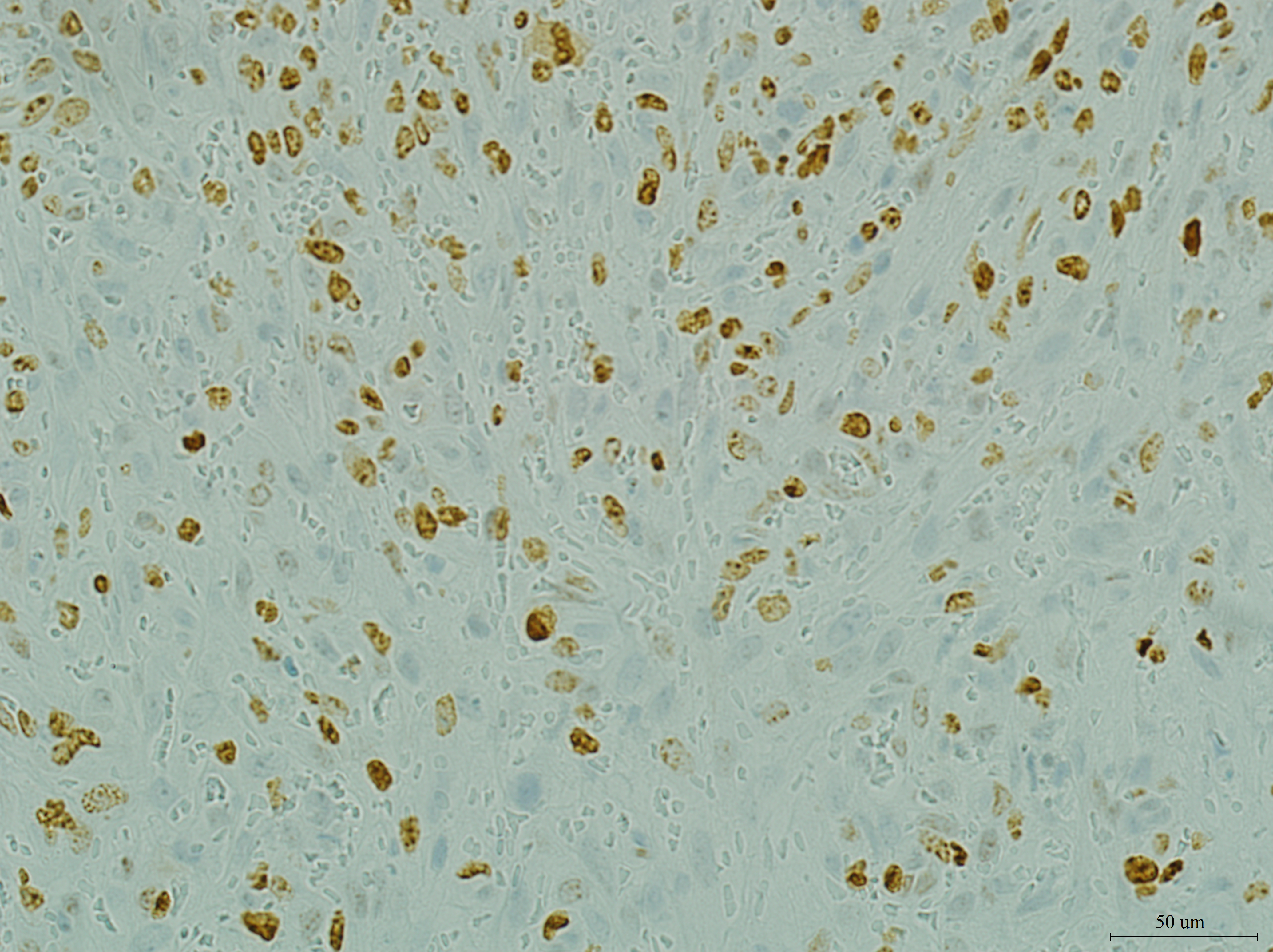

Supplement: S3 Fig — (TIF) [file pone.0145565.s003.tif]

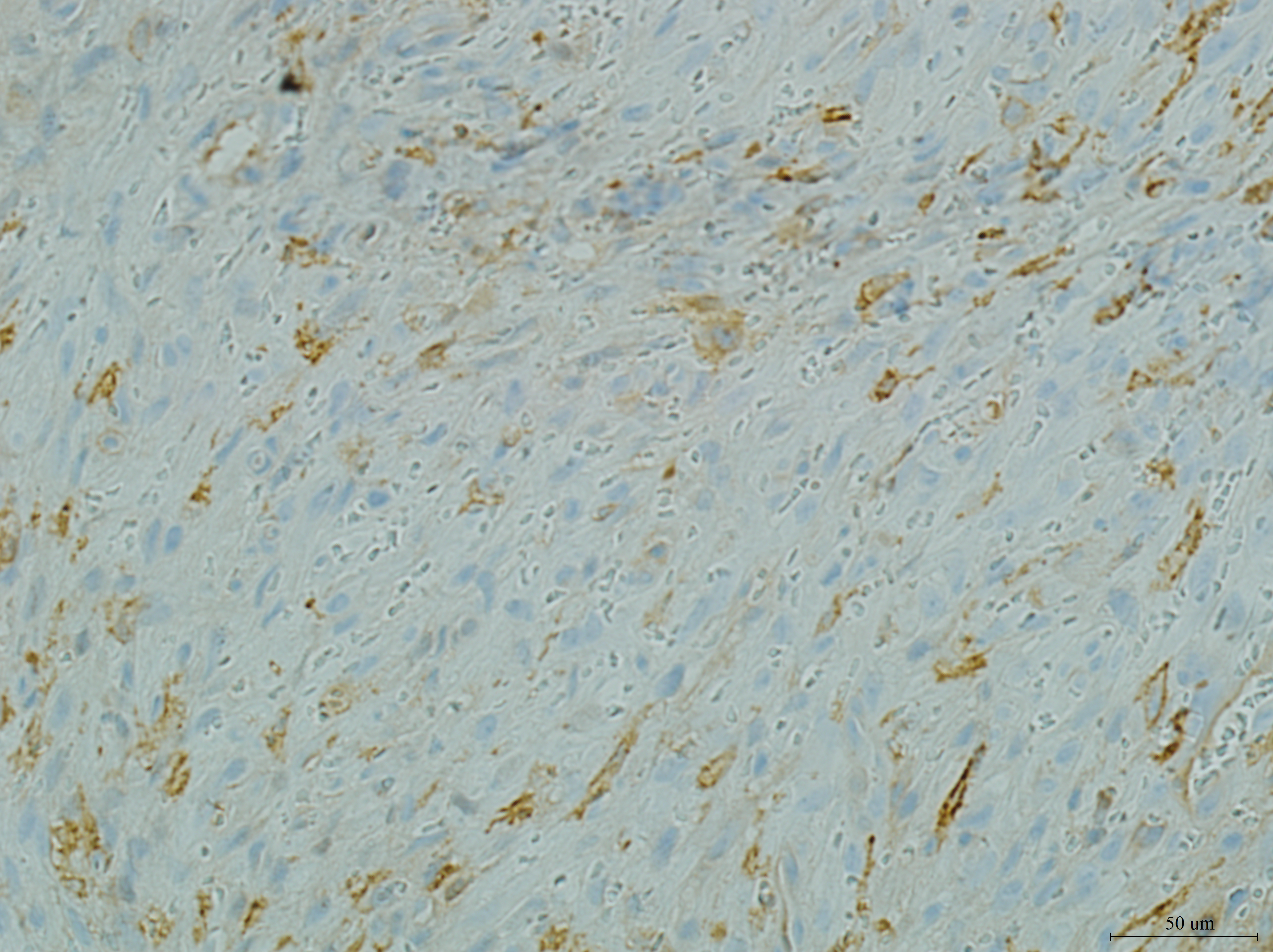

Supplement: S4 Fig — (TIF) [file pone.0145565.s004.tif]

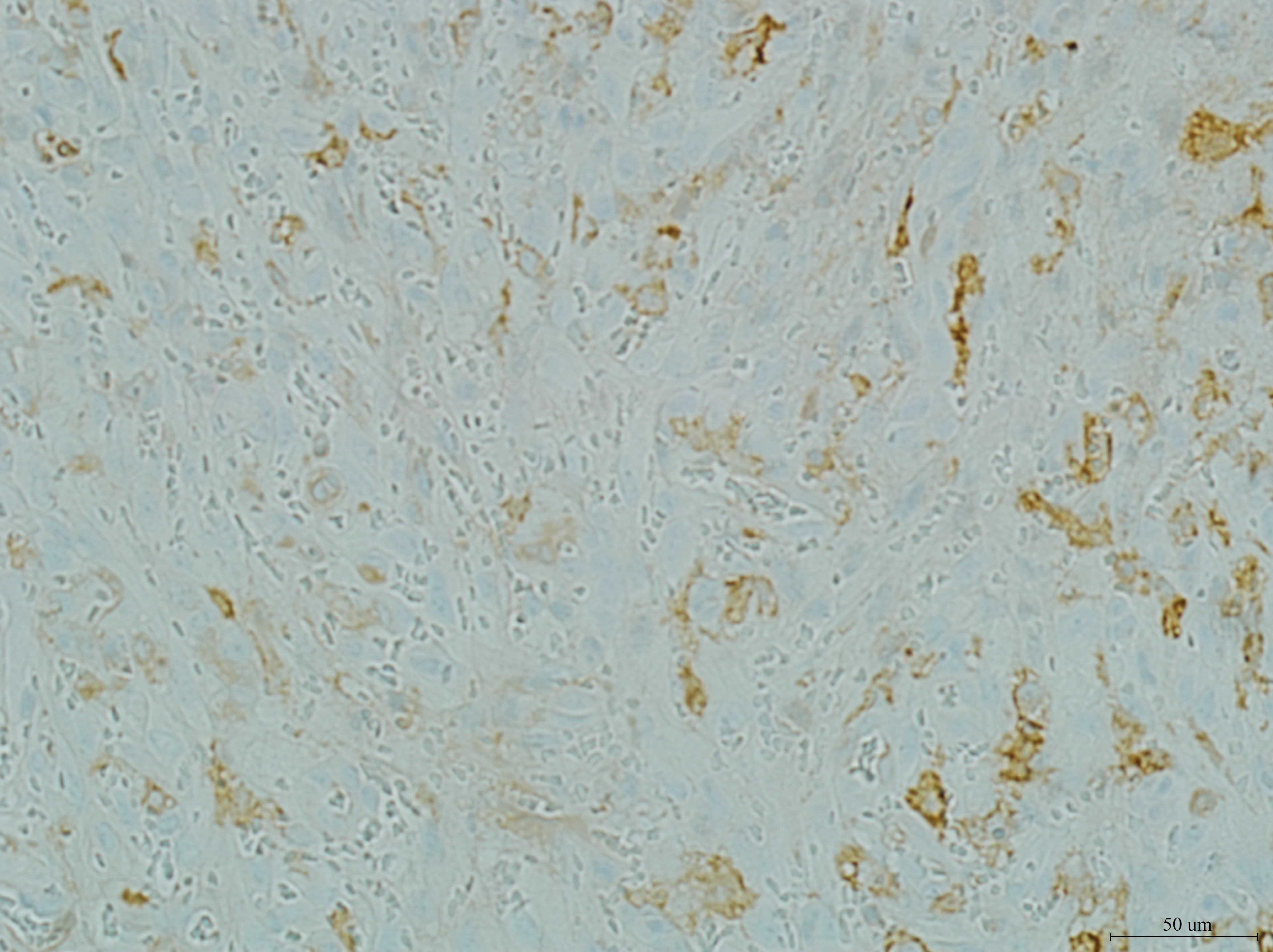

Supplement: S5 Fig — (TIF) [file pone.0145565.s005.tif]

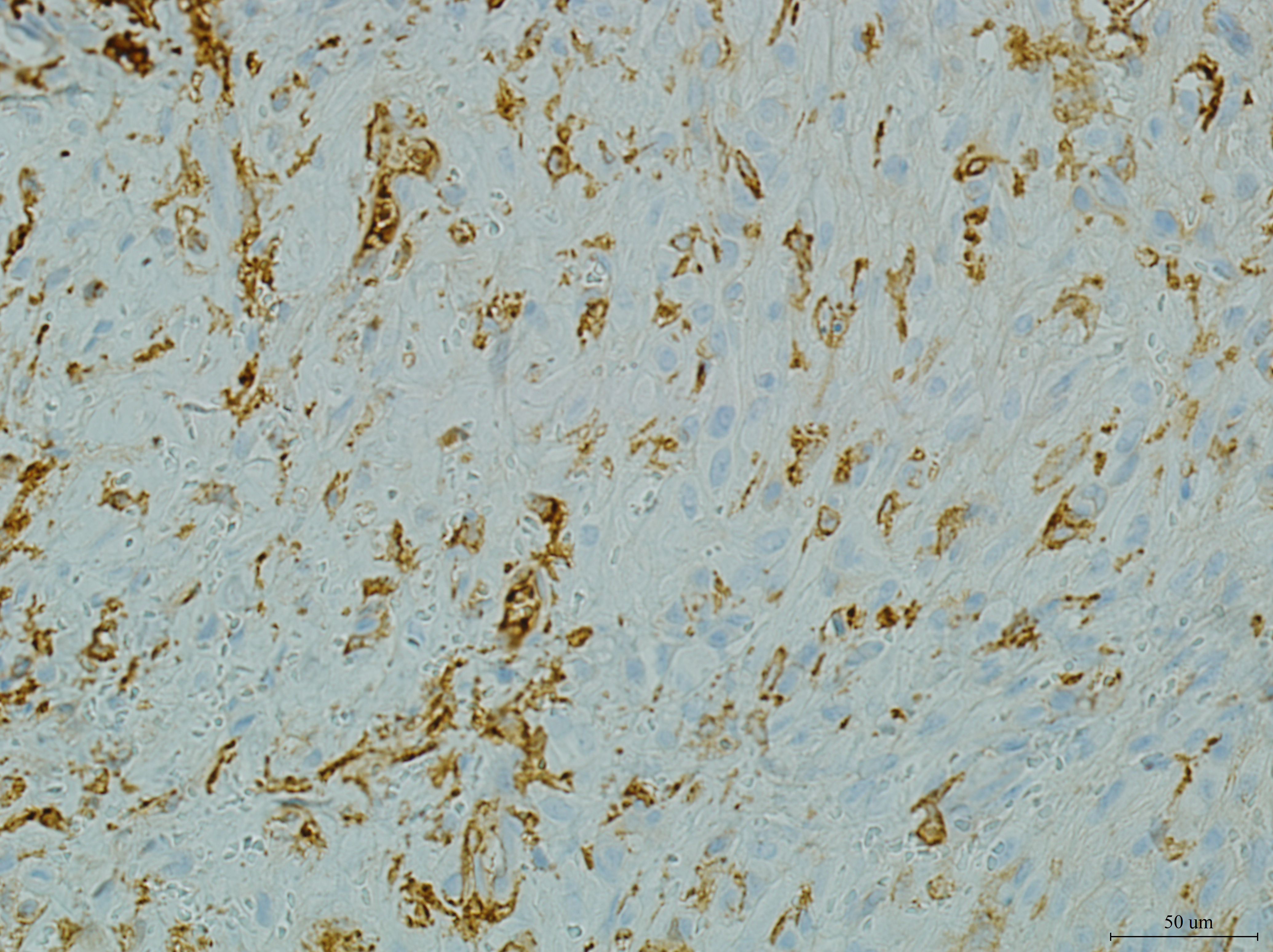

Supplement: S6 Fig — (TIF) [file pone.0145565.s006.tif]
